# Hepatic portal venous gas and bacteremia after colonic endoscopic submucosal dissection: A case report

**DOI:** 10.1002/deo2.107

**Published:** 2022-04-01

**Authors:** Akira Tomioka, Kazuyuki Narimatsu, Nanoka Chiya, Hiroyuki Nishimura, Yoshihiro Akita, Masaaki Higashiyama, Shunsuke Komoto, Kengo Tomita, Ryota Hokari

**Affiliations:** ^1^ Division of Gastroenterology National Defense Medical College Hospital Saitama Japan

**Keywords:** bacteremia, colon cancer, complication, endoscopic submucosal dissection, hepatic portal venous gas

## Abstract

Hepatic portal venous gas (HPVG) is considered to be a sign of poor prognosis in abdominal diseases and a potentially fatal condition. However, HPVG after colonic endoscopic submucosal dissection (ESD), is an even rarer complication that there is just one report of it at the moment. In this report, we present a case of HPVG and bacteremia that happened a day after colonic ESD in the descending colon. A 79‐year‐old female was referred to perform endoscopic treatment for a 40‐mm elevated tumor in the descending colon and surgery for clinical T1b cancer in the rectosigmoid colon. With a preoperative diagnosis of intramucosal carcinoma in adenoma, we performed ESD using carbon dioxide insufflation. The tumor was resected en bloc without any adverse events including perforation. On the following day, shivering and a fever of 38°C suddenly developed with no abdominal symptoms. Computed tomography revealed the presence of HPVG and gas in the middle colic vein without pneumoperitoneum. The patient was managed conservatively with fasting and intravenous antibiotic treatment. We confirmed the disappearance of the findings with computed tomography on the next day of the first computed tomography and with a colonoscope, we observed the base of ESD ulcer 5 days post‐ESD. HPVG might be treated conservatively, but it might cause more severe conditions such as air embolism, so this rare complication still needs to be thoroughly monitored.

## INTRODUCTION

Colonic endoscopic submucosal dissection (ESD) is one of the treatment options for large benign colorectal tumors and early colorectal cancer, but there can be technical difficulties. For instance, having limited endoscopic maneuverability because of the thin walls of the colon. They could lead to a higher risk of adverse events such as bleeding and perforation. Hepatic portal venous gas (HPVG) is a rare complication associated with colonic ESD. The first case of HPVG following colonic ESD was reported in 2018.^1^ This report presents a case of HPVG and bacteremia after colonic ESD performed by an expert for early colon cancer in the descending colon. These complications happened a few hours after oral ingestion. This case was reported following a successful clinical course in which HPVG was managed conservatively and observation of the mucosal defect could be done after ESD, without any adverse events.

## CASE REPORT

**FIGURE 1:**
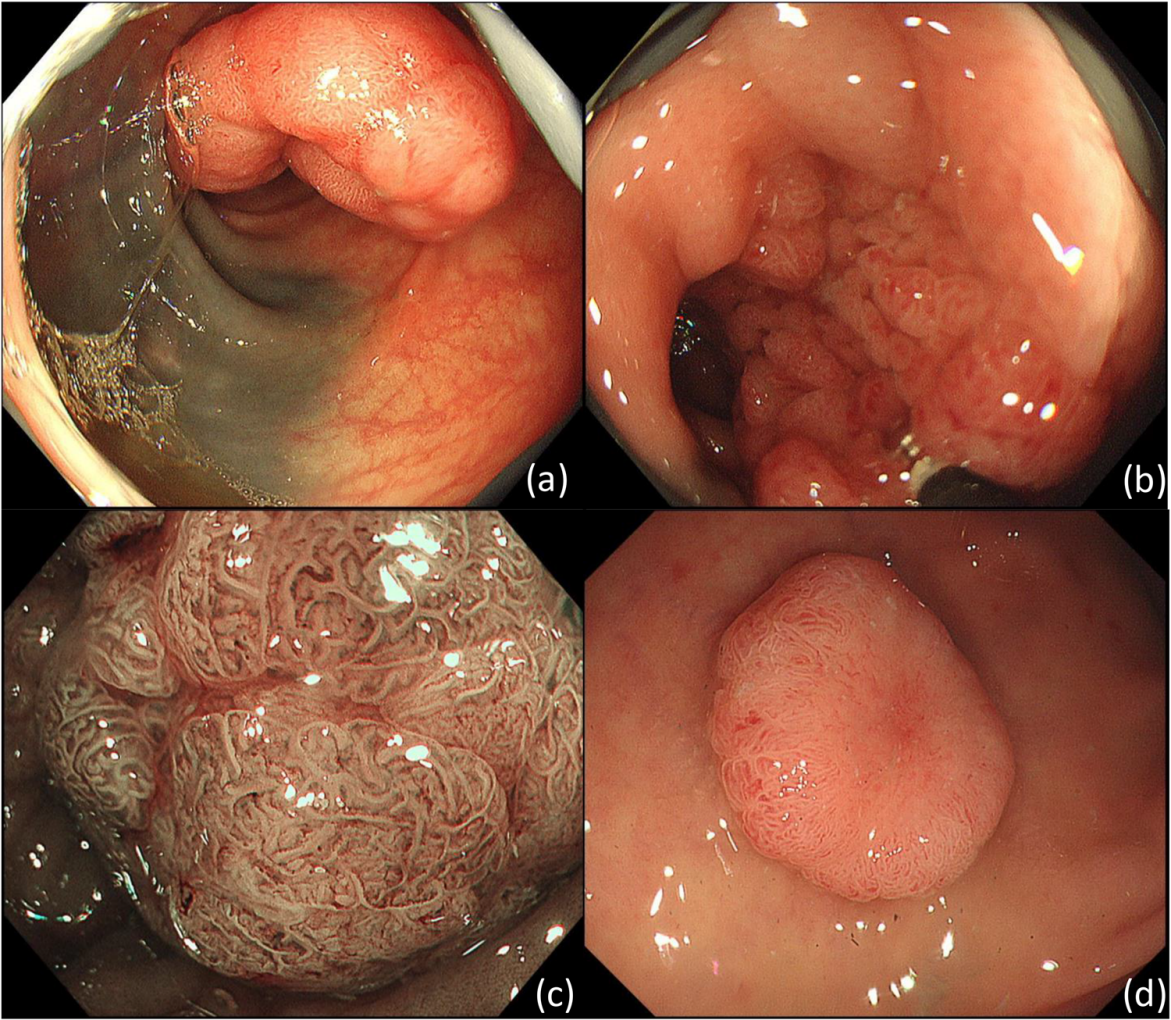
Diagnostic colonoscopy view revealing a 40‐mm sessile lesion in the descending colon (a–c) and a tumor in the rectosigmoid colon (d). (a) The lesion and endoscopic tattoo on the opposite wall were seen. (b) The sessile lesion accompanied by the superficially elevated lesion (type 0‐Is+IIa in the Paris classification). (c) The image of the center of the lesion was observed by magnifying endoscopy with narrow‐band imaging evaluated as Japan Expert Team type 2B. (d) The tumor in the rectosigmoid colon was diagnosed as a deep submucosal invasive carcinoma

**FIGURE 2:**
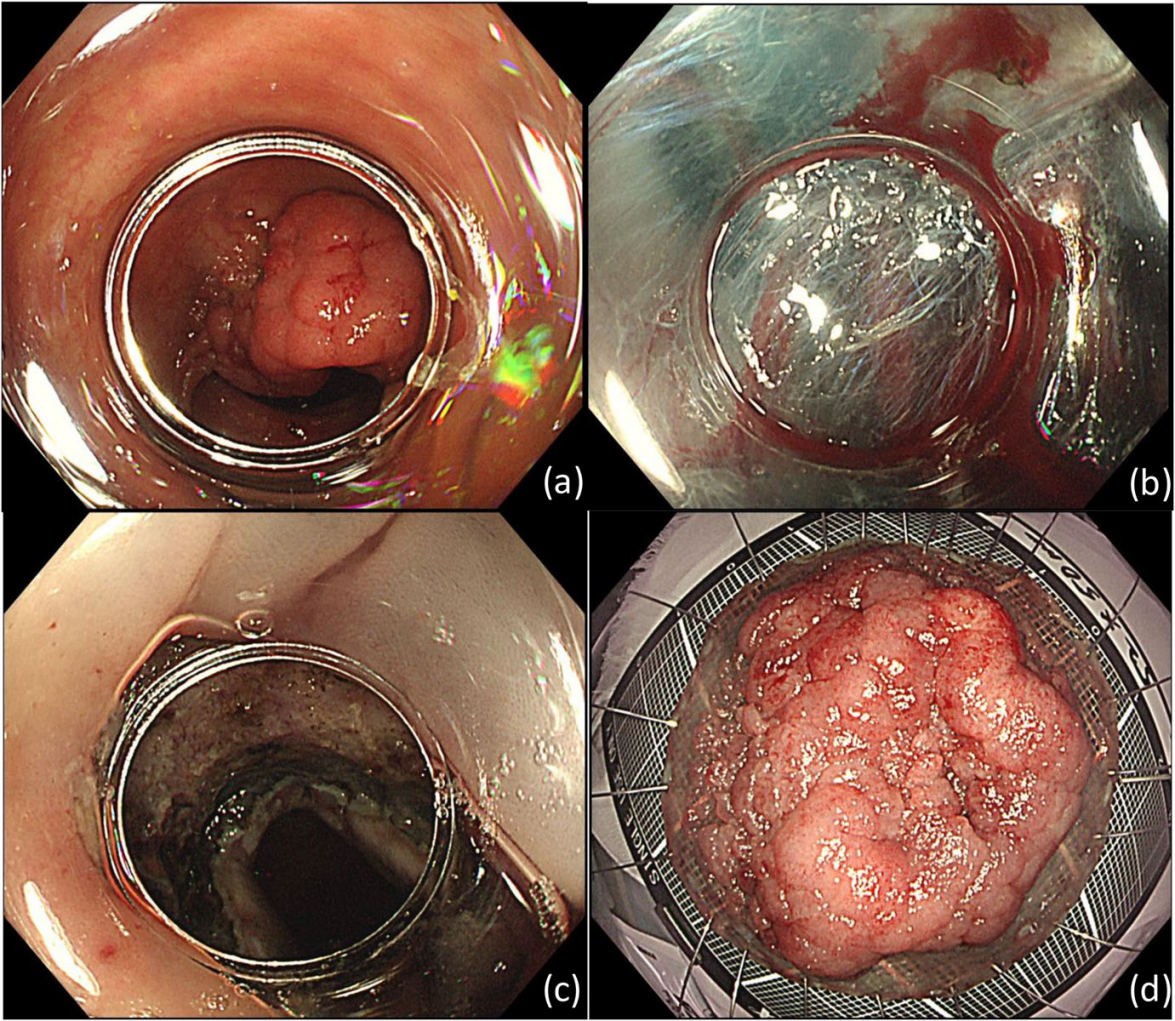
Endoscopic submucosal dissection. (a) We used a tapered‐tip distal attachment cap to facilitate submucosal entry. (b) This tumor had many thick vessels, which were coagulated successfully with hemostatic forceps. (c) This image was taken after exposed blood vessels in the ulcer bed were coagulated. No apparent damage to the muscular layer was seen. (d) The resected specimen was examined pathologically to be intramucosal carcinoma in adenoma

A 79‐year‐old female tested positive for fecal occult blood at a health screening. She had multiple medical comorbidities, including diabetes mellitus, hypertension, and chronic atrial fibrillation. She had a history of cholecystectomy 24 years ago because of cholelithiasis. She underwent colonoscopy (CS) at a local hospital, which revealed a 40‐mm elevated epithelial tumor in the descending colon and a 20‐mm elevated epithelial tumor in the rectosigmoid colon. At our hospital, the tumor in the descending colon was diagnosed endoscopically as an intramucosal carcinoma in adenoma and the tumor in the rectosigmoid colon as a deep submucosal invasive carcinoma after a detailed CS examination (Figure 1). We selected ESD for the tumor in the descending colon after obtaining informed consent. The tumor was resected en bloc using carbon dioxide insufflation without any adverse events including perforation. During ESD, we observed that this tumor had many thick vessels, which were coagulated successfully with hemostatic forceps before dissection. Neither perforation nor damage to the muscular layer was observed after ESD, therefore we did not conduct prophylactic clip closure of the mucosal defect (Figure 2). The ESD procedure time was 72 min. The resected specimen was later examined pathologically, which was intramucosal carcinoma in adenoma.

**FIGURE 3:**
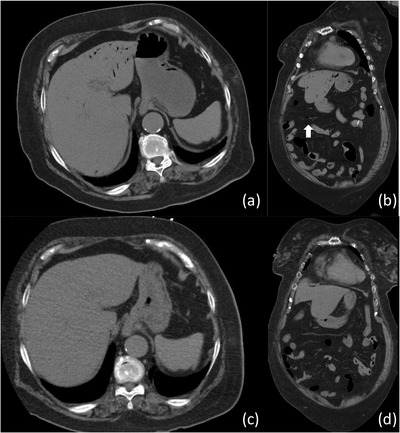
(a, b) Computed tomography revealed the presence of hepatic portal venous gas (HPVG) and gas in the middle colic vein (arrow) without pneumoperitoneum. (c, d) a follow‐up computed tomography showed disappearance of HPVG and gas in the middle colic vein

The following day, peripheral blood tests showed slight inflammation with a white blood cell count of 10,200/μl, and a C‐reactive protein of 2.2 mg/dl which is consistent with ESD reaction. At that point, she did not have any symptoms. Therefore she was permitted to start eating lunch. Later that same day, she reported experiencing shivers and had a fever of 38°C in the evening. There were no abdominal symptoms. Computed tomography (CT) revealed the presence of HPVG and gas in the middle colic vein without pneumoperitoneum (Figure 3a,b). Two bottles of blood culture were obtained, and both showed positive results. The isolated microorganism was Escherichia coli. Based on her symptoms without any peritoneal irritation and no findings of perforation on CT, and since deep mural injuries were not suggested, intravenous antibiotic treatment was started under fasting conditions.

Two days post‐ESD, a follow‐up CT was performed, and it showed the disappearance of HPVG (Figure 3c,d). However, septic shock developed, so we started administering noradrenaline to keep her blood pressure stable.

**FIGURE 4:**
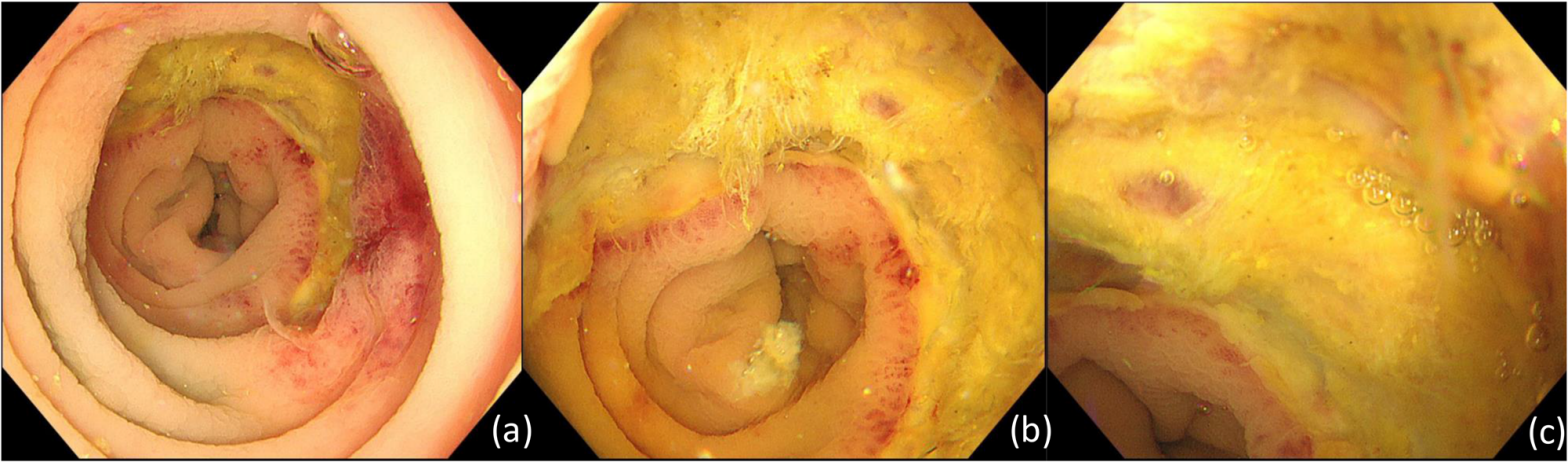
Follow‐up colonoscopy with water immersion technique 5 days post‐endoscopic submucosal dissection . (a) Far view, (b) middle view, and (c) near view

Four days post‐ESD, her condition has improved, so we discontinue noradrenaline administration. Five days post‐ESD, we had not observed any peritoneal irritation signs, but hematochezia was observed. Peripheral blood tests showed drops in hemoglobin levels. Considering any insufflation might make her condition worse, we performed CS with a water immersion technique to stop the bleeding. We observed blood clots on the tumor in the rectosigmoid colon, but the bleeding stopped spontaneously. We also observed the mucosal defect after ESD in the descending colon, which did not have any damage to the muscular layer or the exposed blood vessels (Figure 4). Since we could confirm the findings, oral ingestion was resumed and the patient was discharged eight days post‐ESD.

## DISCUSSION

HPVG is a rare complication associated with colonic ESD, but there is just been one report about this complication.^1^ In addition to our case, both cases could be conservatively treated. The mechanism for HPVG is still unclear, but the main factors to develop HPVG are intestinal wall alteration, bowel distention, ischemia, and sepsis.^2^ In our case, we could confirm the mucosal defect after HPVG developed, which showed no damage to the muscular layer or the exposed blood vessels. HPVG and bacteremia developed about 25 hours after ESD and 3 hours after oral ingestion. This indicates that these complications might not have been caused by the endoscopic procedure itself, but by the mucosal defect and high luminal pressure induced by colonic peristalsis after oral ingestion.

Bacteremia after endoscopic procedures such as endoscopic mucosal resection and endoscopic submucosal dissection rarely happens, even when the mucosal defect is extensive.^3^ Bacteremia might be caused by a result of direct inoculation of bacteria into the submucosa by a contaminated injection needle or direct contact of the exposed submucosa with luminal bacteria. In our case, shivering and a fever of 38°C suddenly developed 3 hours after oral ingestion. Blood culture showed a positive result and the microorganism was Escherichia coli. What this indicates is that there was a low possibility for contamination of blood culture, and it might have happened at the same time with HPVG. Since she had not had any abdominal symptoms and we could also confirm no findings of perforation on CT, these suggest that HPVG and bacteremia were not caused by severe mural damage such as delayed perforation, but caused by sudden inoculation of bacteria and luminal gas into the blood stream from the mucosal defect. Prophylactic clip closure of mucosal defects and antibiotic prophylaxis might be able to reduce these rare complications,^4,5^ considering the pathogenic mechanisms. However further large‐sample studies are needed to conclude how to manage these adverse events.

In conclusion, we report a case of HPVG and bacteremia following colonic ESD. HPVG could be treated conservatively, but it might have caused more severe conditions such as air embolism, so this rare complication still needs careful follow‐up.

## CONFLICT OF INTEREST

The authors declare that they have no conflict of interest.

## FUNDING INFORMATION

None.

## ETHICS STATEMENT

All procedures followed have been performed in accordance with the ethical standards laid down in the 1964 Declaration of Helsinki and its later amendments.

## HUMAN/ANIMAL RIGHTS

All procedures followed have been performed in accordance with the ethical standards laid down in the 1964 Declaration of Helsinki and its later amendments.

## INFORMED CONSENT

Informed consent was obtained from the patient for being published in this case report.
